# Mr. Walker's Introductory Lecture to His Course of Physiology

**Published:** 1808-08

**Authors:** 


Medical and Physical Journal.

VOL. XX.] AUGUST 1808. [NO. 114.

*Printed for R. PHILLIPS, by W. Thorne, Red Lion Court, Fleet Street, London*

### MR. WALKER'S INTRODUCTORY LECTURE TO HIS COURSE OF PHYSIOLOGY;

### EXHIBITING THE NATURAL ARRANGEMENT WHICH HE HAS PROPOSED FOR THAT SCIENCE, AND CONTAINING SOME OF ITS FIRST PRINCIPLES.

*Delivered at Edinburgh in May* 1808.

THE functions of man are the most interesting objects of science. Physics and morals are equally involved by them. It is in them that the physical sciences terminate, and that the moral sciences have their origin.

Of what avail are the former but to develope the influence of external bodies upon the functions of man? What are the latter, but the results of the reaction of that influence upon men in society?

To explain them is the business of physiology; and if it have hitherto failed in doing so; if it have even neglected the noblest of all the functions, I should say the noblest branch of philosophy, the intellectual functions of man, and have abandoned them to persons totally incapable of developing them, because totally ignorant of the structure on which they depend, and who have left them in a state of greater obscurity than that in which they found them, that is the fault of physiologists and not of their science.

In these Lectures, I shall at least be influenced by the deepest sense of such errors.

But it is not merely the philosophy of the human mind, or rather of the intellectual functions of man, that have thus been abandoned to barbarism; I shall endeavour to shew that it is by the aid of physiology alone, that in explaining the functions of the eye, we can render perfect the theories of colour and of vision; that, in explaining the functions of the ear, we can construct rational ones of phonics and of hearing; that, in explaining the functions of

 the the organ of voice, we can illustrate the formation of letters and the origin of languages; that, in explaining the functions of the muscles, we can establish those first principles of taste in the fine arts, the very existence of which have so often been questioned, and so deliver a theory of movement and of attitude applicable to sculpture, the higher species of painting, gesture in oratory, &c.; in fine, it is by physiological means only, that we can construct a natural theory of medicine, and render precise and easy the most delicate and the most difficult of the operations of surgery.

To effect these purposes, nothing is more necessary than arrangement, because it at once involves facts and their theory, or the reasonings founded upon them.

In considering a natural arrangement of Anatomy and Physiology, it appears to me an unquestionable point, that it ought to indicate, at a single glance, the relation of all the functions to, and their dependence upon, each other. It is upon this principle that I have constructed the natural arrangement which I am about to detail; and a single remark on the arrangements adopted by the most celebrated modern Physiologists will, at once, shew the originality of the plan which I propose, and point out the errors of arrangement, which I deprecate.

Blumenbach, Chaunier, Dumas, and Bichat, treat of circulation before absorption, on which it depends, and even before mastication, deglutition and digestion, on which, in general, even absorption itself chiefly depends. Thus, by arranging effects in the place of causes, they confound the relations of the functions, and reverse the very order of their dependence.

If the arrangement of Chaunier in particular who, in this respect, is as accurate as any of them, were to be considered as indicating the relation and dependance of the functions, so absurd is it, that absorption, instead of the cause, would be the result of nutrition, generation the result of absorption, and digestion the result of generation.

The division of the functions into external and internal, as anciently proposed by Aristotle, and afterwards successively adopted and improved by Buffon, Grimaud and Bichat (the last of whom substituted for them the terms organic and animal,) is still more faulty. No class of the functions is either exclusively external or internal. Impressions from external objects are without, but the operations of mind are within; circulation is within, but cutaneous absorption is without.

Anatomy,

Anatomy, I divide into three parts; namely, that which considers the mechanical or loco-motive organs; that which considers the vital organs; and that which considers the intellectual organs.

Under the mechanical or loco-motive organs, I class, first, the bones which support the rest of the animal structure; second, the ligaments which unite them; and third, the muscles which move them.

Under the vital organs, I class, first, the organs of digestion, the absorbent surfaces and the vessels which absorb from these surfaces; second, the heart, lungs, and blood vessels, which derive their contents (the blood) from the absorbed lymph; and third, the organs of secretion, which separate various matters from the blood. Then follow the generative organs, which, though commonly formed into a distinct class from the vital, ought not, by any means to be so reckoned. For it is evident that they form, as it were, the sequel of the vital organs, being dependant on secretion, the last of the vital functions, and destined merely to propagate vitality, and to communicate it to a new series of beings.

Under the intellectual organs, I class first the organs of sense, where impressions take place; second, the brain or organ of judgement, where these excite ideas; and third, the nerves, where volition results from the last.

This is a natural arrangement of the anatomy of animals, and its peculiar simplicity is illustrated by its involving, in its application, that of minerals and vegetables, and by its being capable of instant adaption to physiological science.

In order to arrange Animal Physiology, it is only necessary to substitute the term "Functions" for "Organs", and that science will likewise involve, in application, the physiology of mineral and vegetable bodies, and be, in its term, capable of instant adaption to medical science.

Thus the functions also are divided into mechanical, vital, and intellectual.

The mechanical functions are sub-divided into that of support, that of connection, and that of loco-motion.

The vital functions are divided into that of absorption, that of circulation, and that of secretion.

The intellectual functions are divided into that of sensation, that of mental operation, and that of volition.

A circle of functions, I may observe, thus exist in animals which exist not in minerals or vegetables, because volition, the last of the intellectual functions, connects itself to the mechanical ones by rendering them subservient to it in loco-motion. Thus the first and the last of these actions are as intimately connected as any of the intermediate ones, and a beautiful circle of organic function and influence is formed.

In order to arrange Medical Science, for the term "Healthy Functions", the subject of physiology, it is only necessary to substitute the term "Diseased Functions."

The classes of disease are, therefore, like those of anatomy and physiology, three, namely Diseases of the Mechanical or Loco-motive Functions, Diseases of the Vital Functions, and Diseases of the Intellectual Functions.

The orders of the first class, as affecting the functions of the bones, the ligaments and the muscles, are three, viz. Diseases of Support, Diseases of Connection, and Diseases of Loco-motion.

Those of the second class, as affecting the functions of the absorbent, circulating, and secreting vessels, are likewise three; viz. Diseases of Absorption, Diseases of Circulation and Diseases of Secretion.

Those of the third class, as affecting the functions of the organs of sense, of the brain and of the nerves, are also three; viz. Diseases of Impression, Diseases of Judgment, and Diseases of Volition.

The genera, under each order, consist of Diseases of diminished, irregular and increased functions.

This arrangement of disease likewise involves, in application, that of minerals (if we choose to maintain the analogy, and to give that term to mere injury of structure) and of vegetables, as well as animals. The first have what may be termed Diseases of Mechanical Organs; the second have Diseases of Mechanical and Vital Functions; whilst the third have Diseases of Mechanical, Vital, and Intellectual Functions; corresponding with the observation, that minerals exist; vegetables exist and live; and animals exist, live, and think.

Precisely in the same way would I class the articles of the Materia Medica; first, as operating upon the mechanical, vital, or intellectual organs; and then as either diminishing, rendering regular, or increasing their action.

In this natural arrangement, it will be observed, that all its parts correctly correspond and beautifully flow from each other; while anatomy is the basis of physiology, physiology, in its turn, is the basis of medicine; the classification of one is applicable to the rest, and all of them at once involve mineral, vegetable, and animal nature.

Such arrangement, as it lays the foundation of a new system system of each, will, I trust, soon shed some light over anatomical, over physiological, and particularly over the dark paths of medical science. But it must be remembered, that this exhibits merely a sketch of the application of my General Arrangement to Medical Science, and that sketch necessarily brief, and deprived of every illustration. Yet even after this sketch, I think I may add, in the words of Lord Bacon, "that the harmony of a science, supporting each part the other, is, and ought to be the true and brief confutation and suppression of all the smaller sort of objections."

The Plan of the Course will therefore be precisely the same with that which I have proposed for Anatomy and Physiology in general.

I. In that part of the Course which relates to the mechanical organs and functions, will be explained, the structure and uses, 1st. of the bones which support the rest of the animal machine; 2d. of the ligaments which unite it; and, 3dly, of the muscles which move it.

II. In the part of the Course which relates to the vital organs and functions, will be explained the structure and uses; 1st, of the organs of mastication, deglutition, and digestion of the absorbent surfaces, and of the vessels which absorb from these surfaces; 2dly, of the heart, lungs, and blood vessels, which derive their contents (the blood) from the absorbed lymph; and, 3dly, of the organs of secretion, which separate various matters from the blood. Throughout this division, in particular, the analogies from vegetables will be explained.

III. In that part of the Course which relates to the intellectual organs and functions will be explained the structure and uses; 1st, of the organs of sense, which receive impressions from external objects; 2dly, of the brain, where these impressions excite ideas; and, 3dly, of the spinal marrow and nerves, where volition results from the last. In this division will be also explained, as founded upon original observations, certain criteria of the various degrees of the sensative, perceptive, and voluntary powers possessed by different animals, as well as the nature of sensation, perception, and volition themselves.

In considering the application, or rather the relation of physiology to philosophy in general, the last mentioned points, with regard to the philosophy of mind, will be particularly dwelt upon.

In considering the application of physiology to the fine arts, the errors in the construction of the antique statues, and those of Michael Angelo, both with regard to the individual muscles supposed to be brought into action, and with regard to a hitherto unobserved principle of attitude, will be carefully pointed out.

In considering the application of physiology to the medical and surgical art, a view of a new, simple, and impressive natural system of medical science in general, and of its first principles as an art, will be exhibited, and rules will also be given for performing surgical operations with the utmost precision and ease, upon the principle of an invariable relation between the arrangement of internal and the situation of certain external parts, which consequently form accurate signs of their situation.

No part of the medical art is at once so difficult and so important as this last; nor has any part of it hitherto been so strangely neglected. The lecturer on anatomy thinks this not his duty, because it is of too practical a nature; and the lecturer on medicine thinks it not his, because actual demonstration is foreign to his business. Thus has a part, by far the most important of all medical science, been completely abandoned.

Every other department of medical science may also be more easily studied in private than this individual part of it possibly can, because every other department of it is incomparably more independant of actual demonstration. In short, the utter impossibility of any gentleman who has not practised in this way, ever performing surgical operations with precision and ease, is too obvious to be for a moment dwelt upon.

I think that an exposition of the common errors, a clear explanation of a few original and fundamental principles, and the actual performance of operations upon these principles, will decidedly effect these important purposes.

It is of importance to state, that, by means of this arrangement, notwithstanding the shortness of the Course, not one important fact of anatomy, which can form a basis for physiological reasoning, nor any part of physiology itself, will be omitted.

By adopting this arrangement, therefore, we shall not merely be able to detail a greater number of facts in anatomy, even during a three months course, than can otherwise be in one of six, but we shall be able to deliver all the still more interesting reasonings of physiology, which, though, in general, almost entirely neglected, are more important both to the surgeon and the physician. In truth, anatomy is valuable only as it constitutes their basis.

As,

As, in physiological inquiries, comparative anatomy and comparative views alone can destroy an hypothesis or transmute it into a theory, I shall, throughout the Course, introduce all its principal facts.

The Lectures will consequently consist of Three Parts, each department of physiology being preceded by a view of all the important corresponding facts of human and comparative anatomy, and succeeded by references to their pathological application, in such a manner, as at once to render obvious to the student the facts of anatomy, to impress upon him the important reasonings of physiology, and to exhibit to him a view of a new, simple, and impressive natural system of pathological science in general.

I shall also endeavour to render these Lectures interesting to the student in general science, as well as of medicine; and, in order to facilitate this object, I shall not introduce in them any theory of physiology, until a clear and satisfactory view has been given of the facts of anatomy, on which it may depend.

I may now observe, that the series of beings which the arrangement I have just delivered involves, all, more or less, evidence the phenomena of life and intellect as well as exhibit structure, and may conclude with a few observations concerning these phenomena.

Minerals have properly been said to exist; plants to exist and live; animals to exist, live, and think.

These are all modifications of each other, as I shall afterwards show; but at present, I shall limit my observations to the two last of these classes, and to the additional phenomena; namely, those of life and intellect, which they more strikingly exhibit.

I shall not, however, like some of the most celebrated modern physiologists, occupy my preliminary Lectures, with long details concerning their nature; but, as I reckon life to be the mere result of organization, I shall, throughout the Course, illustrate it in connexion with those organs on which it depends, and shall, at present, only take a general view of their nature.

Life is a term which some philosophers have hitherto inaccurately employed as expressing an imaginnary principle, though it merely designates a series of dependent actions. This they were, doubtless, induced to do by that artifice which has ever induced weak men to assume an air of knowledge, by giving a particular name to what they did not understand; and the same artifice still induces such persons to blame those who endeavour to explain the phenomena of life according to obvious physical laws, precisely because a successful explanation would at once exhibit their ignorance and their desire to conceal it.

Life has, with equal absurdity, been termed a forced state, though it merely evidences a series of phenomena in justest unison with those of the universe. I dissent therefore from those who assert that we employ the terms *life*, and *vital force*, to express what are at least apparent exceptions to general laws. This is only a repetition of the error of Brown. It was only, however, an erroneous conclusion from accurate observations.

"For example," says Cuvier, "let us contemplate a female in the prime of youth and health. That elegant voluptuous form; that graceful flexibility of motion; that gentle warmth; those cheeks crimsoned with the roses of delight; those brilliant eyes, darting rays of love, or sparkling with the fire of genius; that countenance enlivened by sallies of wit, or animated by the glow of passion, seem all united to form a most fascinating being. A moment is sufficient to destroy this illusion. Motion and sense often cease without any apparent cause. The body loses its heat; the muscles become flat, and the angular prominences of the bones appear; the lustre of the eye is gone; the cheeks and lips are livid. These, however, are but preludes of change still more horrible. The flesh becomes successively blue, green, and black. It attracts humidity; and while one portion evaporates in infectious emanations, the other dissolves into a putrid sanies, which is also speedily dissipated, In a word, after a few short days, there remains only a small number of earthy and saline principles. The other elements are dispersed in air and in water, to enter again into new combinations."

This, he adduces as a proof, that the possession of life prevents the operation of external physical causes. The picture is perfectly correct, but the conclusion unauthorised.

It was, indeed, impossible for us not to feel the frequent necessity of deploring the early fate of those we loved. We, therefore, thought we perceived this principle operating, when the evident influence of external causes, or some mysterious agency, seemed to drive the noblest results of life, intellect and genius, from the philosopher and statesman, who had, by the diffusion of truth, and resistance to arbitrary power, preserved the liberties, and aggrandized the happiness of nations; we thought we perceived their influence influence when imagination's powers fled from the poet, whose song had once almost conferred new life on the heroes of ancient times; we thought we perceived their influence when the phenomena of vital action disappeared in the body of the patriot hero, in whom they had once developed the noblest specimen of that generous love of social life which induced him to devote his existence to his country's weal; we thought we perceived their influence when those magic charms abandoned the lovely female, whose powers had once excited even from the philosopher those splendid epithets that ranked her with the other objects of his adoration, celestial truth and godlike genius; which had obtained even from the poet those wonderful strains that at once immortalized him and the object of his song; which had commanded even in the hero those glorious devotions which, for far too short a period, illustrated the ever memorable days of chivalry.

When those eyes, whose soft languor had fascinated every beholder; those cheeks, where life had often spread the crimson veil of love: when these were gone, those eyes were dim and sunk, those cheeks were pale, those lips were livid, that bosom depressed, that form rigid and icy cold: when all had thus assumed the darkest and most frightful hues, had gradually lost cohesion, and become one mass of horrible putrescence, where infectious effluvia now threatened destruction, almost welcome, to those who once adored the animated being,—it was scarcely possible to feel all this and avoid the conclusion that life was a state of force.

It was doubtless this unknown and imaginary power connecting the various phenomena of organic structure, that gave origin to the idea of life. But that they are no state of force is proven by this, that while the only notion we have of them is motion,—successive attraction and repulsion, electricity, fire, &c. exhibit precisely corresponding phenomena; and the last of these was, I believe, on that single account, considered as the symbol of life and intellect by the great Zoroaster.

This and similar facts convince me, that the earth has, myriads of times, been illumed by the highest attainments of intellect, and as often plunged in darkness. This single point of Indian philosophy was, in my opinion, but the mere shadow of the highest excellence in philosophy in general; and, as the greatest cultivation of the human mind, and the greatest misapplication of its faculties, cannot well exist together, I am equally convinced, that the beautiful beautiful specimens of art, blended with heterogeneous absurdities in the monuments of Upper Egypt, afford the most striking proof, that, contrary to the common assertion, there *were* periods in that country when tyrants held no despotic sway over the physical, nor *false* priests over the moral structure of man.

But to return to the subject. True it is, that the affinities of external agents with the molecules of organic bodies are precisely the same during life, when their influence is resisted, as after death when it operates with the greatest energy; but the power which, during life, organic bodies possess of resisting the influence of external agents, and even the extension of the sphere of their action beyond the mere bounds of the living body, is, in my opinion, very easily accounted for.

All the particles of matter possess attraction and repulsion, and the largest homogeneous masses, cæteris paribus, evidence them most. Hence organic bodies, while they posess homogeneity by the subsistence of intimate relations between their parts, evidence attraction and repulsion in the highest degree. While, in infancy, these relations are merely forming, the powers of attraction and repulsion are weak; when, in the vigor of life, these relations are most intimate, these powers are greatest; when, in old age, these relations are interrupted, these powers are also diminished; and when, in death, these relations cease to exist between the molecules, or, in other words, when the body is no longer organized, the power of resistance terminates.

Thus life is no forced state, but an intrinsic power arising merely from the intimate relations of these molecules; and the greater effects which, in such combination, they are capable of producing, than when, in death, they have become independent of each other, and their powers, disunited, are capable of much less effectual resistance.

Thus it is that I would endeavour to show a connexion between the phenomena of living bodies and the general laws of nature.

This general and common motion of all the parts, says Cuvier, forms so peculiarly the essence of life, that the parts which are separated from a living body soon die, because they possess no motion of their own, and only participate in the general motion produced by their union. Thus, according to the expression of Kant, the mode of existence of each part of inanimate bodies belongs to itself, but, in living bodies, it resides in the whole. When

I say

I say that life consists in motion, I make no assertion derogatory to the Deity, for every philosopher is of opinion that motion is to us as mysterious as life. At all events, it would be dishonest of me to give my sanction to opinions respecting a difference which seems to me not to subsist. The same causes which terminate motion terminate life. Life begins by motion, and terminates when it ceases; but very curiously it also terminates in consequence of it.—In other words, the motion wears out the machine in which it takes place. For, while the functions are most dependant, life increases; and their very independence, though, to a certain extent, the grand aim of existence, destroys it. The very cause of decay commencing when the different functions have acquired the greatest vigour, arises precisely from their having acquired that vigour, and being, at that moment, most capable of a species of warfare, if I may so term it, with each other, as well as, in some measure, from the greater number of accidents to which a complexity of functions is liable.

That, at this period, the functions may, in a certain sense, be said to combat each other, will be evident to every one who, for a moment, considers, that great exercise of the loco-motive functions uniformly interrupts the vital and intellectual, or of the vital functions injures the loco-motive and intellectual, or of the intellectual functions injures the loco-motive and vital.

*These powers are always in an inverse relation to each other.*

Grimaud, says Richerand, has given the most extensive development to this idea, of the constant opposition between these two series of actions, over which preside, according to the opinion of this physician, two powers, which he calls loco-motive and digestive. It is marked in no species of animals more than in the carnivorous, which connect with senses abounding in stratagem, and muscles capable of extraordinary action, an assimilating power of so low a degree, that their aliment, in order to be conveniently digested, must possess a composition analogous to that of their organs.

Thus, in my opinion, the independence which the functions acquire, and the interruptions which their operations present to each other, subjects them to the common laws of impeded motion and its result—a separation of parts. When, in infancy, their unison is most complete, life is most vigorous. When in old age, this unison ceases, life disappears. Hence also I am convinced it is, that those animals whose functions are longest dependent on each other, other, and who remain longest helpless, longest enjoy organic existence.

As motion only can result from motion, so life can only result from life. The vital actions of all animals originate in those of their parents, from whom they are detached, precisely when their actions become sufficiently independent of them. I am convinced, it is upon this principle, that the egg is detached from the ovary, and the fœtus from the uterus, as well as because, in these cases, the ovary and the uterus themselves, after long quiescence, recover an independent action of their own. The same power which enables an organic body to act by itself, enables it to separate from another.

Life then seems in a great measure to consist in the successive attraction and repulsion of particles—properties equally common to minerals, vegetables, and animals, though in different manners and degrees. Life, however, is not here to be confounded with sensibility, which nevertheless, I shall afterwards shew is also possessed by all of them. Life seems unseparable from generation, nutrition, and decay, or, in other words, from origin, increase, diminution—a progression which minerals and vegetables pass through as well as animals. Minerals increase by additions to their external surfaces, vegetables by absorbing from without inward, and animals by absorbing from their internal as well as external surfaces

